# Microglial response to increasing amyloid load saturates with aging: a longitudinal dual tracer in vivo μPET-study

**DOI:** 10.1186/s12974-018-1347-6

**Published:** 2018-11-06

**Authors:** Tanja Blume, Carola Focke, Finn Peters, Maximilian Deussing, Nathalie L. Albert, Simon Lindner, Franz-Josef Gildehaus, Barbara von Ungern-Sternberg, Laurence Ozmen, Karlheinz Baumann, Peter Bartenstein, Axel Rominger, Jochen Herms, Matthias Brendel

**Affiliations:** 1 Department of Nuclear Medicine, University Hospital, LMU Munich, Marchioninistraße 15, 81377 Munich, Germany; 2 German Center for Neurodegenerative Diseases (DZNE) Munich, Feodor-Lynen-Str. 17, 81377 Munich, Germany; 3 Roche, Pharma Research and Early Development, NORD DTA / Neuroscience Discovery, Roche Innovation Center Basel, F. Hoffmann-La Roche Ltd., Grenzacherstrasse 124, CH-4070 Basel, Switzerland; 4 Department of Nuclear Medicine, Inselspital, University Hospital Bern, Freiburgstrasse 4, 3010 Bern, Switzerland; 5 Center of Neuropathology and Prion Research, Feodor-Lynen-Straße 23, 81377 Munich, Germany; 6 Munich Cluster for Systems Neurology (SyNergy), Munich, Germany

**Keywords:** TSPO μPET, Amyloid μPET, Alzheimer’s disease, Neuroinflammation, Microglia, Aging

## Abstract

**Background:**

Causal associations between microglia activation and β-amyloid (Aβ) accumulation during the progression of Alzheimer’s disease (AD) remain a matter of controversy. Therefore, we used longitudinal dual tracer in vivo small animal positron emission tomography (μPET) imaging to resolve the progression of the association between Aβ deposition and microglial responses during aging of an Aβ mouse model.

**Methods:**

APP-SL70 mice (*N* = 17; baseline age 3.2–8.5 months) and age-matched C57Bl/6 controls (wildtype (wt)) were investigated longitudinally for 6 months using Aβ (18F-florbetaben) and 18 kDa translocator protein (TSPO) μPET (18F-GE180). Changes in cortical binding were transformed to *Z*-scores relative to wt mice, and microglial activation relative to amyloidosis was defined as the *Z*-score difference (TSPO—Aβ). Using 3D immunohistochemistry for activated microglia (Iba-1) and histology for fibrillary Aβ (methoxy-X04), we measure microglial brain fraction relative to plaque size and the distance from plaque margins.

**Results:**

Aβ-PET binding increased exponentially as a function of age in APP-SL70 mice, whereas TSPO binding had an inverse U-shape growth function. Longitudinal *Z*-score differences declined with aging, suggesting that microglial response declined relative to increasing amyloidosis in aging APP-SL70 mice. Microglial brain volume fraction was inversely related to adjacent plaque size, while the proximity to Aβ plaques increased with age.

**Conclusions:**

Microglial activity decreases relative to ongoing amyloidosis with aging in APP-SL70 mice. The plaque-associated microglial brain fraction saturated and correlated negatively with increasing plaque size with aging.

## Background

The progressive accumulation of senile plaques composed of β-amyloid (Aβ) is a main pathological hallmark of Alzheimer’s disease (AD), the most common dementing disorder in the elderly. The Aβ accumulation promotes synaptic loss and neuronal degeneration apparently by activating microglia, the resident macrophages of the brain [1–4]. In the healthy brain, microglia cells are long-lived cells using highly motile processes to survey parenchymal territory for the presence of pathogens and cell debris. In addition, microglia secrete factors that support neuronal survival and synaptogenesis [5]. In the early stages of AD, microglia migrate towards amyloid deposits and express certain cell-surface receptors to promote the clearance and phagocytosis of Aβ [6–8]. Furthermore, deficits in microglia activation favor accelerated amyloid deposition [9]. However, it has been hypothesized that microglial reactions are overwhelmed by the massive Aβ deposition in later AD stages [10, 11]. This suggestion is supported by the finding that plaque-associated microglia ultimately show decreased expression of Aβ-binding receptors, which leads to a significant reduction in Aβ degradation by microglia in the aging brain [11]. Moreover, plaque-associated microglial cells show a threefold higher mortality rate compared to non-plaque-associated microglia in vivo [12].

The use of small animal positron emission tomography (μPET) with Aβ tracers enables longitudinal investigations of cerebral amyloidosis in rodents in vivo [13, 14]. Confirmation of the hypothesis of a ceiling effect in microglial reactions has been hampered by the technical difficulty in following the fate of aging microglial cells in living mice. The past decade has seen the introduction of Aβ-μPET in rodents [15, 16], using the same radioligands employed in the clinical routine for the differential diagnosis of AD [17, 18]. A series of PET radiotracers targeting the microglial marker 18-kDa translocator protein (TSPO), formerly known as the peripheral benzodiazepine receptor (PBR) [19–23], has been developed in recent years.

The basal availability of TSPO binding sites is low in the healthy living brain (21), such that local upregulation presents a sensitive marker for the detection of microglial activation in afflicted brain regions [24–26]. This is supported by findings of elevated TSPO expression in the hippocampus and the frontal, temporal, and parietal cortices of postmortem AD brain [25, 27, 28]. Our group has recently established cross-sectional dual tracer μPET imaging of Aβ and TSPO in transgenic AD mouse models [29]. Given this background, we aimed in the present longitudinal Aβ/TSPO double tracer μPET study to explore the longitudinal association between amyloidosis and microglial response during aging of an amyloid mouse model in vivo. By using mice with a range of baseline age, we were able to perform correlation analysis with the longitudinal biomarker progression over 7 months. Final immunohistochemistry supported the interpretation of μPET results by mapping of individual plaques and microglial cells.

## Methods

### Animals and study design

All experiments were carried out in compliance with the National Guidelines for Animal Protection, Germany, and with the approval of the regional animal care committee (Regierung Oberbayern) and were overseen by a veterinarian. Animals were housed in a temperature- and humidity-controlled environment with a 12 h light–dark cycle, with free access to food (Sniff, Soest, Germany) and water.

**Fig. 1:**
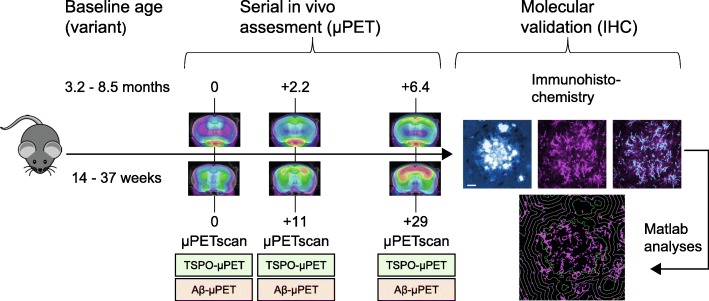
Schematic illustration of the study design. In vivo β**-**amyloid small animal positron emission tomography (Aβ-μPET) and 18 kDa translocator protein (TSPO)-μPET imaging was performed in a longitudinal design with baseline examination at 0 months, follow-up at + 2.2 months, and terminal examination at + 6.3 months in the Alzheimer’s disease (AD) mouse model, APP-SL70. Molecular validation of μPET results was performed via immunohistochemistry in mid- (11.4 to 12.7 months) and late-aged (13.6 to 15.3 months) APP-SL70 mice after the final μPET-scan. Custom-written Matlab software was used to quantify histological results

All experiments were performed in APP-SL70 mice, a mouse-line produced by Roche (Basel, Switzerland), (*N* = 17, baseline age: 3.2 to 8.5 months of age: 3.2 to 5.0 months (*N* = 4); 5.1 to 6.8 months (*N* = 6); and 6.9 to 8.5 months (*N* = 7)). First fibrillar Aβ deposits in this mouse-line appear as early as 2.5 months of age, similar to the mouse line used by Blanchard [30]. Congophilic plaques are observed starting from 5 to 6 months of age. Protein levels of Aβ40 and Aβ42 start to increase from 3 months of age and range around 1 ng/mg brain at 6 months, 25 ng/mg brain at 9 months, and 90 ng/mg brain at 12 months of age. μPET examinations (Aβ and TSPO) were performed in a longitudinal design at baseline (0 months), follow-up (+ 2.2 months/11 weeks) and terminal age (+ 6.3 months/29 weeks). Serial μPET scans of both tracers deriving from a total of 30 age-matched C57Bl6 mice (wt) served as control data. All mice were killed after terminal scanning, followed by rapid brain removal and performance of immunohistochemistry analyses. The study design is illustrated in Fig. 1.

### Radiochemistry

Radiosynthesis of [^18^F]-GE180 was performed as previously described [23], with slight modifications [29]. This procedure yielded product with radiochemical purity exceeding 98%, and molar activity of 1400 ± 500 gigabecquerel (GBq)/μmol at end of synthesis. Radiosynthesis of [^18^F]-florbetaben was performed as described earlier [16, 31].

### μPET data acquisition, reconstruction and preprocessing

All μPET procedures followed an established standardized protocol for radiochemistry, acquisition, and post-processing [29, 32]. Mice were anesthetized with isoflurane (1.5%, delivered at 3.5 l/min) and placed in the aperture of the Siemens Inveon DPET (Siemens, Knoxville, USA), as described previously [33]. In brief, we made TSPO-μPET emission recordings during 60–90 min (p.i.) per image of [^18^F]-GE180 (11.2 ± 1.5 megabecquerel (MBq)) beta-amyloid-μPET emission recordings during 30–60 min p.i. of [^18^F]-florbetaben (10.8 ± 1.5 MBq). All images were spatially normalized using automatic algorithms and tracer specific templates [32].

### μPET data analyses

All μPET analyses were performed with PMOD (V3.5, PMOD technologies, Basel, Switzerland). Normalization of emission images to standardized uptake value ratio (SUVR) images was performed using a previously validated white matter reference region [29, 32]. A target volume of interest (VOI) was placed in the bilateral frontal cortex (46 mm^3^) and SUVR_CTX/WM_ values were extracted for each individual APP-SL70 and wt mouse at the serial imaging time points for both tracers. Differences of TSPO- and Aβ-μPET SUVR between terminal and baseline time points were calculated as percentage change (Δ%). *Z*-scores of the TSPO- and Aβ-μPET tracer uptake in individual APP-SL70 mice were calculated by normalization to age-matched wt mice. To this end, the mean uptake in age-matched wt mice was subtracted from the individual value in APP-SL70 mice (APP-SL70_INDIVIDUAL_ - wt_MEAN_) and the resulting difference divided by the corresponding standard deviation for wt mice to generate an individual *Z*-score value.

$$ Z-\mathrm{score}=\frac{\mathrm{APPSL}70\left(\mathrm{INDIVIDUAL}\right)-\mathrm{wt}\left(\mathrm{MEAN}\right)\ }{\mathrm{wt}\left(\mathrm{SD}\right)} $$

To establish a readout of microglial activity relative to fibrillary amyloidosis we calculated *Z*-score differences between TSPO- and Aβ-μPET. *Z*-scores deriving from the same imaging time point of individual mice (gap 0.35 ± 1.62 weeks).

$$ Z-\mathrm{score}\ \mathrm{difference}=Z-\mathrm{score}\ \left( TSPO-\upmu \mathrm{PET}\right)-Z-\mathrm{score}\ \left( A\beta -\upmu \mathrm{PET}\right) $$

### Immunohistochemistry: acquisition and image analysis

Brains intended for immunohistochemistry were fixed by immersion in 4% paraformaldehyde at 4 °C for 15 h. Two representative 50-μm-thick slices per animal were then cut in the axial plane using a vibratome (VT 1000 S, Leica, Wetzlar, Germany). Free-floating sections were permeabilized with 2% Triton X-100 overnight and blocked with I-Block™ Protein-Based Blocking Reagent (Thermo Fischer Scientific, Waltham, USA). We obtained immunofluorescence labelling of microglia using an Iba-1 primary antibody (Wako, Richmond, USA) with a dilution of 1:200 in I-Block™ and the A-21244 secondary antibody (Invitrogen, Carlsbad, USA) with a dilution of 1:500 in I-Block™. For histological staining against fibrillar Aβ, we used methoxy-X04 (TOCRIS, Bristol, United Kingdom) with a dilution of 0.01 mg/ml. The unbound dye was removed in three washing steps with PBS, and the slices were then mounted on microscope slides with fluorescent mounting medium (Dako, Santa Clara, USA). Images were acquired with a LSM 780 confocal microscope (Zeiss, Oberkochen, Germany) equipped with a 40x/1.4 oil immersion objective. The excitation wavelength for Iba-1 detection was 633 nm and emission was detected from 638 to 755 nm. For methoxy-X04, the excitation wavelength was 405 nm and emission was detected from 403 to 585 nm for each brain slice. We acquired three-dimensional 16-bit data stacks of 2048 × 2048 × 120 pixels from five different positions in the frontal cortex at a lateral resolution of 0.17 μm/pixel and an axial resolution of 0.4 μm/pixel. To quantify Iba-1 positive brain volume fraction, hereinafter referred as microglia brain fraction, as well as plaque density and size, we utilized custom-written Matlab software (MathWorks, Natick, USA). The detailed method was described previously [34].

Local background subtraction was used to diminish intensity variations between different stacks. Subsequently, microglia cells were identified by applying the 90th percentile as minimal-intensity threshold. Noise was excluded by applying a connected component analysis excluding patches of contiguous voxels smaller than 1 μm^3^. Analyses were performed by an operator who was blind to the μPET results.

### Statistics

The associations between μPET readouts (Δ%, *Z*-score, *Z*-score difference) and age were characterized by applying linear, logarithmic, and quadratic regression analyses as implemented in SPSS (SPSS Version 24, IBM SPSS Software, IBM, Armonk, New York). In cases with several statistically significant fits (*p* < 0.05), the best curve fitting model was determined by applying the Akaike Information Criterion (AIC) [35]. If the AIC proved indifferent between two models, we chose the one with the higher *R*^2^ value. Statistics of histological analyses were calculated in Prism 7.01 (GraphPad Software, San Diego, CA, USA). Statistical comparison of the microglia fraction between different plaque radii was performed for the highest microglia occupancy in the vicinity to the plaque border [6]. Data was tested for normal distribution using the D’Agostino and Pearson omnibus test. Intergroup comparisons were performed using the two-tailed unpaired Student’s *t* test. For correlation of plaque size and microglia brain fraction, the variables were compared across groups using one-way analysis of variance (ANOVA). All specifications of n state the number of biological replicates. All results are presented as mean ± standard error of mean (SEM).

## Results

### Microglial response saturates relatively to ongoing amyloidosis during aging

**Fig. 2:**
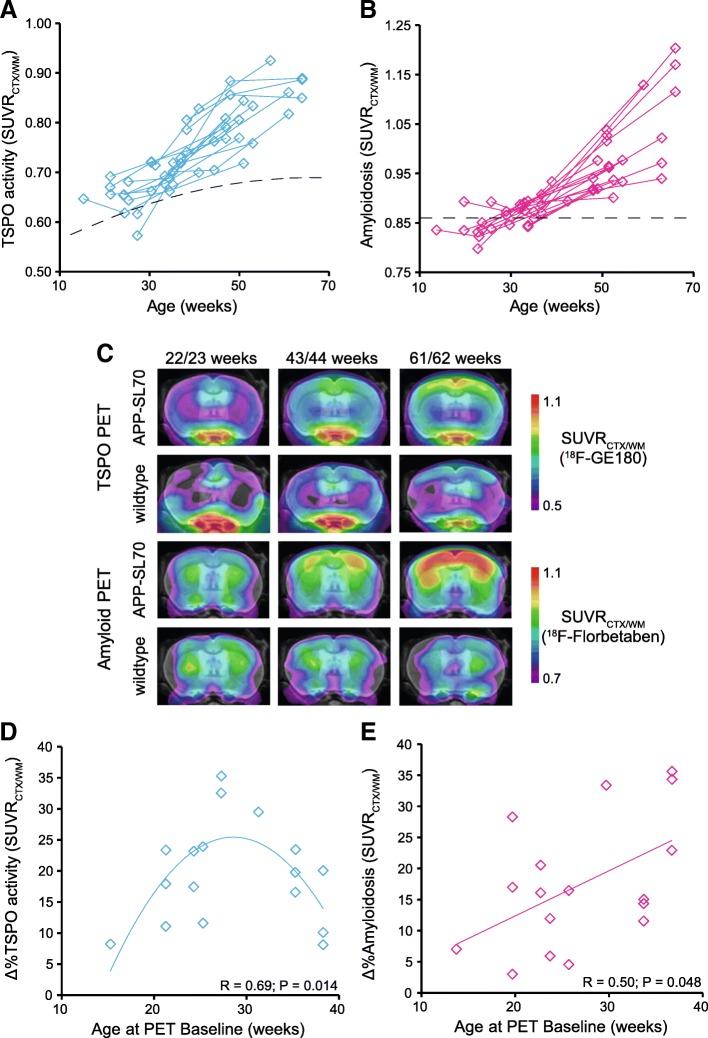
Microglial response increased but saturated relative to ongoing amyloidosis during aging. Plots show cortical Standardized Uptake Value Ratio (SUVR) of [^18^F]-GE180 (TSPO-activity) (**a**) and [^18^F]-florbetaben (amyloidosis) (**b**) in APP-SL70 mice at different ages (B) APP-SL70 indicate increasing cortical amyloidosis and 18 kDa translocator protein (TSPO) binding during aging (**c**). Percentage change for both PET tracers between baseline and + 6.3 months as a function of baseline age in APP-SL70 mice and wt reveal an inverted U-shape for TSPO activity (quadratic fit, *R* = 0.69, *p* = 0.014 (**d**) but a linear positive association for amyloidosis in APP-SL70 (linear fit, *R* = 0.50, *p* = 0.048 (**e**). *N* = 17

**Fig. 3:**
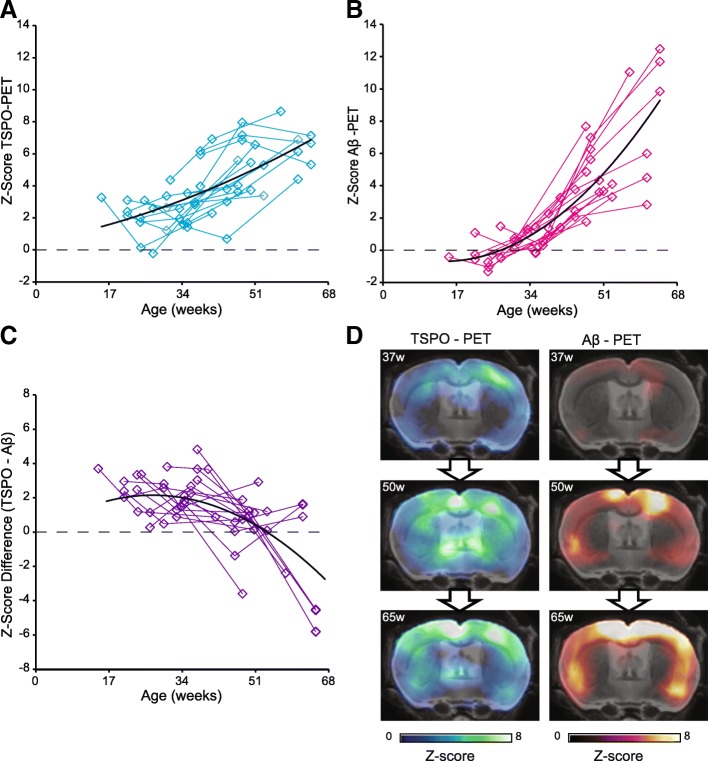
Standardized μPET analysis (*Z*-scores) TSPO activity and amyloidosis. Standardized TSPO activity (**a**; quadratic fit, *R* = 0.68, *p* < 0.001) and standardized fibrillar amyloidosis (**b**; quadratic fit, *R* = 0.86, *p* < 0.001) indicate an increase with aging. The direct comparison of both standardized tracer signals reveals a decrease in the *Z*-score differences (TSPO—Aβ) with aging (**c**; quadratic fit, *R* = 0.66, *p* < 0.001). **d** Exemplary findings of a single APP-SL70 mouse show that microglia response is overwhelmed by ongoing amyloid deposition. *N* = 16

First, we analyzed serial changes of TSPO and fibrillar amyloidosis by dual tracer μPET to characterize the AD mouse model through molecular imaging. Both TSPO-μPET (+ 2.8 ± 2.4% per month) and Aβ-PET signals (+ 2.9 ± 2.5% per month direct comparison of TSPO-PET and Aβ-PET increase rates: *p* = 0.897) increased strongly during aging of individual APP-SL70 mice. At late time points, the two markers were distinctly elevated when compared to wt mice (Fig. 2a–c) and there was a strong direct association between SUVR values of both PET tracers (quadratic fit, *R* = 0.90, *p* < 0.001). However, the percentage change of PET SUVR between baseline and + 6.3 months as a function of starting age in APP-SL70 mice showed an inverted U-shape for TSPO binding (quadratic fit, *R* = 0.69, *p* = 0.014, Fig. 2d) but a linear positive association for amyloidosis (linear fit, *R* = 0.50, *p* = 0.048, Fig. 2e). Thus, increases of microglial activity in aged APP-SL70 mice tended to reach a plateau whereas amyloidosis continued to progress even at late follow-up. Given the differences by tracer in μPET alterations as functions of age, we aimed to compare directly the longitudinal time courses of TSPO activity and amyloidosis. To this end, we calculated standardized *Z*-scores for individual mice and for both tracers based on findings in age-matched wt controls. By this approach, we found the expected strong increases with age for TSPO activity (quadratic fit, *R* = 0.68, *p* < 0.001; Fig. 3a) and fibrillar amyloidosis (quadratic fit, *R* = 0.86, *p* < 0.001, Fig. 3b). Next, *Z*-score differences (TSPO—Aβ) for all serial imaging time points were introduced as a measure of microglial activity relative to fibrillar amyloidosis. Importantly, we observed a decreasing *Z*-score difference as a function of age in APP-SL70 mice (quadratic fit, *R* = 0.66, *p* < 0.001, Fig. 3c), which clearly revealed that the microglial response to ongoing amyloid deposition is relatively attenuated at the later ages ((*Z*-score difference < 0) ≥ 12.2 months of age) of the mouse model. Notably, this effect was not only observed at the group level but was also distinguishable in single animals (Fig. 3d).

### Plaque-associated microglial brain fraction decreases with increasing plaque size during aging

**Fig. 4:**
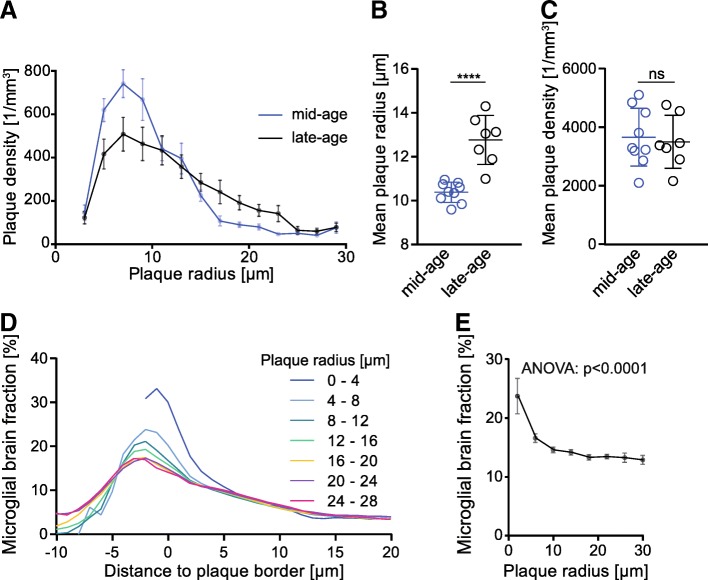
Molecular elucidation of in vivo μPET findings by terminal immunohistochemistry. **a** Frequency distribution of plaque radii in mid-aged (11.4 to 12.7 months) and late-aged (13.6 to 15.3 months) APP-SL70 mice. The mean plaque radius (**b**) is significantly higher in the late-aged cohort when compared to mid-aged APP-SL70 mice (*p* < 0.0001, two-tailed Student’s *t* test), whereas the plaque density (**c**) did not indicate changes during aging > 12 months in APP-SL70 mice (*p* = 0.746, two-tailed Student's *t* test). **d** Correlation of microglial brain fraction with distance to plaque border and plaque size. Each profile represents the change of microglial brain fraction with distance to the border of plaques with defined radius. **e** Microglial brain fraction in the vicinity to the plaque border (radius 1 μm) decreased significantly with increasing plaque radius (one-way ANOVA, *F*(5,16) = 11.87, *p* < 0.0001). Data presented as mean ± SEM; *n* = 7–9 mice

To confirm and extend observed in vivo results, methoxy-X04 staining of fibrillar Aβ as well as Iba-1 staining of microglia were performed in mid- (11.4 to 12.7 months) and late-aged (13.6 to 15.3 months) APP-SL70 mice after the final μPET-scans. In line with previous studies, our immunohistochemical analysis showed that the observed increase in the Aβ-μPET signal is indicative of plaque growth rather than increased plaque density (Fig. 4a, b). In late-aged APP-SL70, mice the mean plaque radius was significantly elevated by approximately 3 μm compared to that in mid-aged mice (*t*_(14)_ = 5.86, *p* < 0.0001, two-tailed Students *t* test, Fig. 4b), whereas plaque density in both age groups remained unchanged at approximately 3600/μm^3^ (*t*_(14)_ = 0.33, *p* = 0.746, two-tailed Students *t* test, Fig. 4c). Furthermore, plaque size distribution analysis showed a shift towards larger radii in late-aged compared to mid-aged transgenic mice (Fig. 4a).

**Fig. 5:**
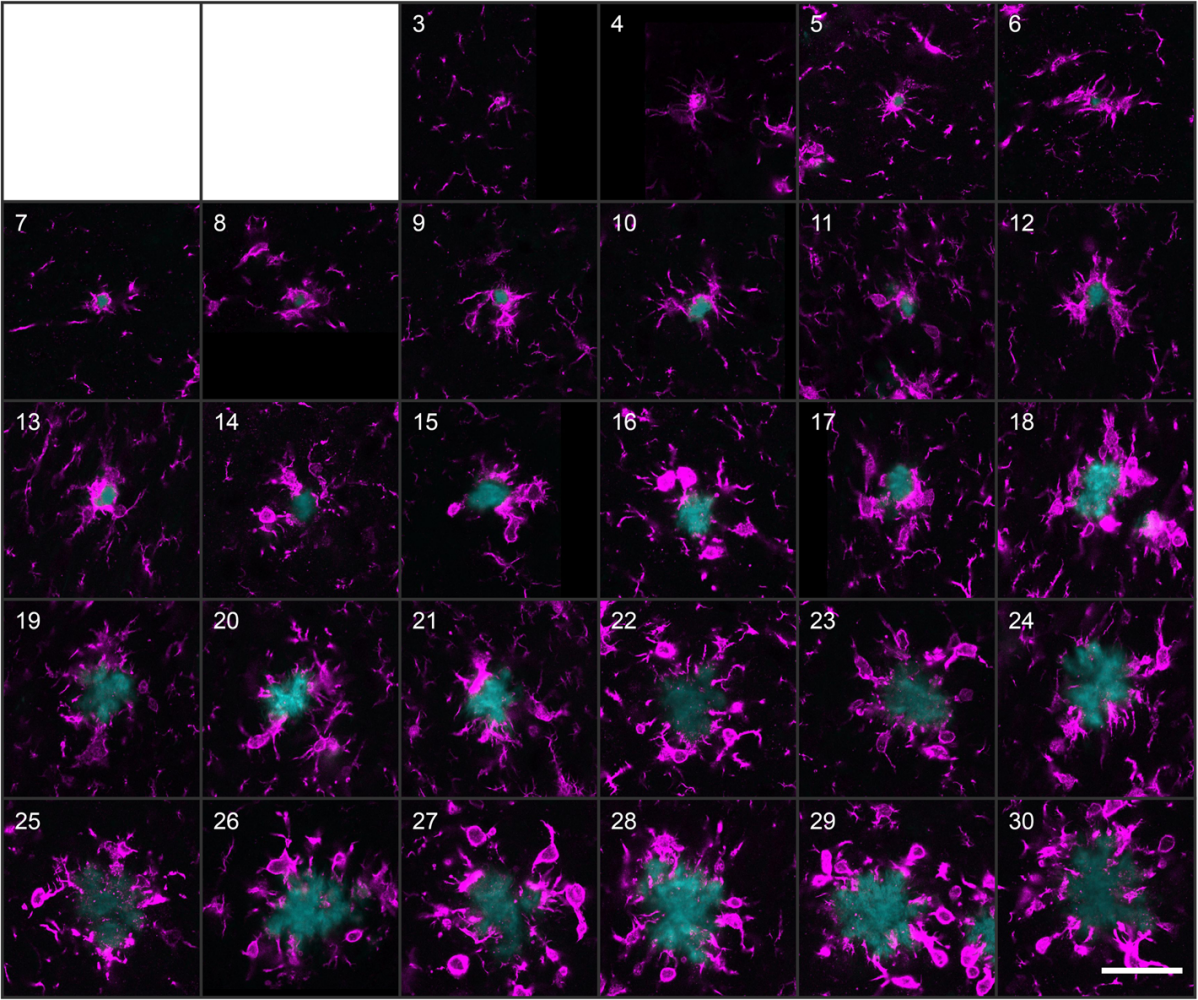
Microglial brain fraction was maximal at small plaques. β-amyloid plaques of radius increasing from 3 to 30 μm are illustrated according to their size (white numeral in upper left corner). Each pane shows Methoxy-X04 stained plaque (cyan) together with the Iba-1 immunosignal (magenta). Scale bar 40 μm

Microglia proliferation in plaque-free areas and their migration towards Aβ plaques has already been shown to occur in AD model mice [12], which results in an increased number of microglial cells surrounding amyloid deposits [36]. However, our standardized μPET analysis revealed a decrease of the TSPO signal in direct relation to the increasing amyloid signal with aging (Fig. 3c). To assess the molecular relationship between plaque growth and microglial brain fraction, we applied custom-written MATLAB cluster analysis for automated morphological detection by applying the 90th percentile as the minimal intensity threshold for identifying microglial cells. We analyzed a total of 1312 plaques ranging in radius from 3 μm to 30 μm and identified their associated microglial cells via immunofluorescence. Representative plaques of several sizes are illustrated in Fig. 5. Interestingly, when we calculated the mean volume occupied by Iba1-positive microglia in consecutive 1-μm-thick layers around the plaque border up to 20 μm distance, we observed a maximum of microglial brain fraction near small plaques (radii between 3 and 4 μm), while the microglia brain fraction decreased with increasing plaque radius (*F*(_5,16_) = 11.87, *p* < 0.0001, one-way ANOVA, (Fig. 4d, e, Fig. 5). We conclude that the observed age-related decrease of the TSPO-μPET signal relative to the Aβ-μPET signal was likely driven by a decrease in the microglial brain fraction around large plaques, which came to predominate in late-aged APP-SL70 mice (Fig. 4b).

### Microglial brain fraction in the plaque-free cortical brain parenchyma of APP-SL70 mice was less compared to wt mice

**Fig. 6:**
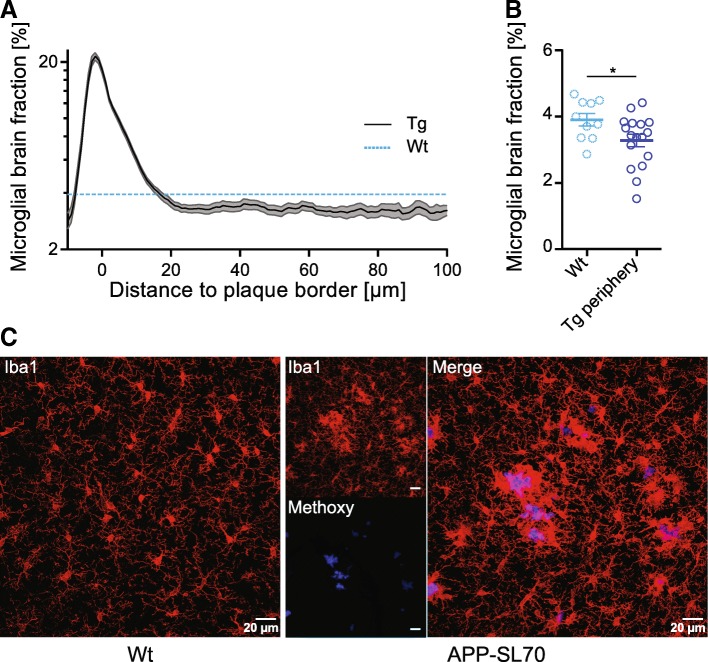
Microglial brain fraction decreased in the plaque-free cortical brain parenchyma of APP-SL70 mice. **a** Microglial brain fraction as a function of the distance to the plaque border in APP-SL70 mice (black line) when compared to the mean microglial brain fraction in wildtype (wt) mice (dotted blue line). **b** Direct comparison of microglial brain fraction of APP-SL70 mice (> 30 μm from plaque borders) and wt mice (mean). Microglial brain fraction is significantly reduced compared to wt mice (*p* < 0.05, two-tailed Student’s *t* test). **c** Iba-1 immunofluorescence staining in a wt mouse aged 16 months in comparison to a double staining of Iba-1 and methoxy-X04 stained plaque in APP-SL70 mouse aged 15 months. Data are presented as mean ± SEM; *n* = 10–17

It is well acknowledged that microglial cells are essential for proper brain function and that microglia can rapidly proliferate in response to a wide range of central nervous system insults [37, 38]. In this context, a recent study of microglial turnover and proliferation in AD showed comparable rates of proliferation and loss for plaque-associated microglial cells indicating a steady-state, while non-plaque-associated microglial cells showed a threefold higher proliferation rate. In contrast, wt mice show only moderate rates of microglial cell proliferation and loss [12]. In the present study, we observed an increase in microglial brain fraction in APP-SL70 mice only in proximity to the plaque border, starting at a maximal distance of 20 μm (Fig. 6a). Surprisingly, we detected a significantly lower microglial brain fraction distant to plaques in the APP-SL70 mice compared to the microglial brain fraction in wt mice (*t*_(25)_ = 2.18, *p* < 0.05, two-tailed Students *t* test, Fig. 6b). It is well-known that microglial cells are activated by Aβ deposits and actively migrate towards the plaque within 1 to 2 days after the initial formation of an amyloid deposit [6, 39]. Regarding the threefold higher proliferation rate of non-plaque-associated microglial cells, the net microglial loss distal to plaques in APP-SL70 compared to wt mice, while surprising, is conspicuous in our double-labelling studies (Fig. 6c).

## Discussion

We present the first longitudinal in vivo dual tracer μPET study aiming to directly compare the time courses of microglial activation and fibrillar amyloidosis with age in a transgenic amyloid mouse model. Our results clearly indicate that both biomarkers increase with age, but that microglial activation is disproportionately elevated at an early age and seems to saturate relative to amyloidosis, which continues to progress. Detailed immunohistochemical analyses revealed a significant decrease of microglial brain fraction around amyloid plaques with increasing plaque radius to be the cellular correlate of our in vivo μPET findings. Moreover, we found that the microglia brain fraction in the plaque-free brain parenchyma of APP-SL70 mice was lower than in wt mice. This depletion of microglial cells distal to plaques is likely related to the massive microglial migration towards zones of fibrillar Aβ deposition [6, 39].

With this serial in vivo study, we aimed to investigate longitudinal relationships between microglial activation and amyloidosis during the life course of the APP-SL70 AD mouse model. We performed dual-tracer small animal μPET examinations with the novel tracer [^18^F]-GE180 for TSPO and [^18^F]-florbetaben for fibrillar amyloidosis, in conjunction with immunohistochemical analyses after the final imaging studies. To enable a reliable comparison of the relationship between the two μPET readouts, we took pains to develop a standard procedure for quantification, entailing a biphasic calculation method: First, we calculated standardized tracer specific *Z*-scores of individual mice at different time points by considering mean and standard deviation values of age-matched wt mice. We next calculated differences between TSPO- and Aβ-μPET Z-scores as a measure of microglial activation relative to fibrillar amyloidosis. We deemed this calculation of a difference score to be more reliable than a ratio method, as values close to zero would potentially have distorted the results at the onset of fibrillar amyloidosis in young mice. The two radioligands have different sensitivities for their specific targets, resulting in distinct detection thresholds unequal magnitudes of signal alterations during the progression of the AD model. To address these issues, we used the standardized *Z*-score calculation as our main endpoint. In fact, our analysis showed positive *Z*-score differences at early ages of APP-SL70 mice, which suggest that microglial activation precedes fibrillar amyloidosis at the onset of amyloid pathology. However, even with standardized *Z*-scores, there remains some possibility that this effect may be related to a higher sensitivity of the TSPO ligand to its target compared to the applied Aβ tracer. In contrast, due to its baseline dependency, the longitudinal decrease of the *Z*-score difference towards late ages is a rather compelling readout unlikely to be biased by possible tracer sensitivity differences. Furthermore, ceiling effects are unlikely as far higher magnitudes of TSPO activation and amyloidosis can be detected with these tracers in other circumstances [31, 40]. Thus, our serial dual tracer μPET imaging proves that microglial activation saturates during an ongoing fibrillar amyloid deposition in this mouse model (Figs. 2 and 3). Our PET findings are absolutely in line with a recently observed plateau during TSPO PET imaging in aged APP23 mice by the same radioligand [41]. The results are also in line with findings for other biomarkers of microglial function, i.e., the peak in sTrem2 levels in cerebrospinal fluid of patients with mild cognitive impairment [42], followed by a drop in patients who have converted to dementia [43]. Even more importantly, computed longitudinal courses of sTrem2 in individuals with dominantly inherited AD decrease after symptom onset whereas amyloid deposition continues to progress [44], thus concurring with the presently observed relations between TSPO expression and fibrillar amyloidosis in aging APP-SL70 mice.

Although an important strength of μPET lies in its fitness for longitudinal monitoring and target quantification, molecular imaging has limitations in spatial resolution and in its applicability for resolving mechanistic processes. For this reason, we supplemented μPET with a detailed immunohistochemical study of activated microglia and histological staining of fibrillar Aβ, which together supported automatized volumetric computations. Our data clearly indicate that microglia fraction adjacent to plaques declines with increasing plaque size (Fig. 4d). Given that plaque size but not density increases with advanced age (Fig. 4b, c), it seems obvious that the microglial activity must decrease relative to fibrillar amyloidosis over time. We validated these findings by comparing mid- and late aged APP-SL70 groups, concluding that the decreasing microglial brain fraction with increasing plaque size is consistent with our μPET results in vivo. Since a recent study showed that brain location of microglia is a relevant factor for its morphological classification [45], our specific analysis of frontal cortical microglia cells in wt and APP-SL70 mice seems appropriate as it matched to the regional PET analysis.

So far, it remains unclear when and why microglial activity decreases adjacent to plaques. It is known that microglia migrate within 1–2 days towards newly formed amyloid deposits, where they promote the clearance and phagocytosis of Aβ by expressing certain cell-surface receptors [6–8]. However, during disease progression, plaque-associated microglial cells show a decrease in fiber mobility [46], lower expression of Aβ-binding receptors [11], and moreover also show a threefold higher mortality rate compared to non-plaque associated microglial cells [12]. Nonetheless, the same in vivo study reported a threefold higher proliferation rate of microglia distal to plaques in AD compared to wt mice, suggesting that new microglial cells migrate from the periphery to the plaque border [12]. However, we observed even lower microglial brain fraction distal to plaques of aged APP-SL70 mice compared to wt animals (Fig. 6). We conclude that the rate of microglia cell migration towards Aβ depositions in APP-SL70 mice eventually exceeds the rate of proliferation of microglia cells staying in the peripheral zone. With aging, this potentially leads to an exhaustion of microglial cells for migration towards Aβ depositions and, together with the increased rate of microglial cell loss around plaques [12], this may explain the observed decrease in microglia brain fraction with increasing plaque radius.

As a limitation of this study, we note that the amyloid tracer does not distinguish soluble and oligomeric Aβ. Thus, we cannot disentangle if there is a stronger association between microglia and soluble or oligomeric proportions of Aβ, which could show different growth rates with aging. Furthermore, given the nature of our longitudinal PET design, we were not able to acquire immunohistochemistry from mice at younger ages but instead focused on the late stage of the disease. Proliferation rates, mortality, and spatial distribution of microglia during their whole life cycle should therefore receive attention in future cross-sectional designs.

## Conclusion

Taken together, findings of this preclinical study in transgenic mice reveal the individual trajectories of microglial activation in relation to Aβ deposition, thus providing important information about the staging of AD-like pathology, which could guide human clinical research. The translation of findings in animal models to human disease is challenging, but the strong bidirectional translational science potential of μPET findings to clinical PET holds great promise to dramatically advance our understanding of AD.

## Abbreviations

AD: Alzheimer’s disease
AIC: Akaike Information Criterion
ANOVA: Analysis of variance
Aβ: β-amyloid
GBq: Gigabecquerel
MBq: Megabecquerel
p.i: Post injection
PBR: Peripheral benzodiazepine receptor
SEM: Standard error of mean
SUVR: Standardized uptake value ratio
TSPO: 18 kDa translocator protein
VOI: Volume of interest
wt: Wildtype
Δ%: Percentage change
μPET: Small animal positron emission tomography
